# The safety and effectiveness of rehabilitation exercises on COVID-19 patients: A protocol for systematic review and meta-analysis

**DOI:** 10.1097/MD.0000000000021373

**Published:** 2020-07-31

**Authors:** Renjun Gu, Senlei Xu, Ziyun Li, Yihuang Gu, Zhiguang Sun

**Affiliations:** a The First School of Clinical Medicine; b School of Acupuncture and Tuina, School of Regimen and Rehabilitation; c The Third School of Clinical Medicine; d The Second School of Clinical Medicine, Nanjing University of Chinese Medicine, Nanjing, Jiangsu Province, China.

**Keywords:** COVID-19, meta-analysis, rehabilitation exercises, systematic review

## Abstract

**Background:**

Corona Virus Disease 2019 (COVID-19) is spreading fast starting late 2019. It mainly affects the human respiratory system. Many reports revealed that rehabilitation exercise can improve respiratory function and relieve the pressure from diseases, but there is no evidence to prove its effects on COVID-19. This protocol of systematic review and meta-analysis will clarify the safety and effectiveness of rehabilitation exercises on different COVID-19 patients.

**Methods and analysis:**

Related studies will be retrieved from Web of Science, Cochrane Library, PubMed, Chinese Biomedical Literature Database, Chinese National Knowledge Infrastructure, Chinese Scientific Journal Database, Wan fang Database, ClinicalTrials, WHO Trials, and Chinese Clinical Trial Registry. Pre-defined inclusion and exclusion criteria will be used by two researchers independently for extracting data from literatures. Clinical recovery time and effective rates will be assessed as the primary outcomes. Changes of patient's condition, pulmonary function, respiratory function and activity of daily living will be assessed as the secondary outcomes. Fixed effect model will be used for evaluating efficiency. Considering clinical heterogeneity, random effect model will be used for continuous outcomes. Funnel charts, subgroup analysis and sensitivity analysis will be used to explore sources of heterogeneity. Endnote X9.3 will be used to manage data screening. The statistical analysis will be completed by RevMan5.2 and Stata/SE 15.1 software.

**Result:**

This study will assess effects and safety for practicing rehabilitation exercises on COVID-19 patients.

**Conclusion:**

The conclusion of this study will provide evidence to prove the safety and effectiveness of rehabilitation exercises on COVID-19 patients.

## 1 Introduction

Coronavirus disease 2019 (COVID-19) is a new acute infectious disease.^[1]^ It attracted great attention of public health. World Health Organization (WHO) has listed it as a public health emergency of international concern. COVID-19 is contagious^[2]^ and human-to-human transmission has been confirmed.^[3]^ It mainly causes fever, fatigue, cough, shortness of breath, etc.^[4]^ Symptomatic treatment and supportive care are currently considered the major treatment methods.^[5]^ Per National Institutes of Health, a guidance on classifying severity of illness is appearing to have 5 levels, asymptomatic or presymptomatic infection, mild illness, moderate illness, severe illness, and critical illness.^[6]^

Rehabilitation exercise uses moderate body movements to help the body return to normal state such as gymnastics, tai chi, qigong, etc. Proper exercises can improve cardiopulmonary function.^[7–9]^ COVID-19 affects the respiratory system and causes dyspnea. Some patients will then develop muscle soreness and symptom of fatigue.^[10]^ As of patients with mild or moderate symptom, rehabilitation exercises improve respiratory system and relieve symptoms of muscle pain. In addition, governments use quarantines to help stopping the spread during COVID-19 outbreaks, and it can also create patients with negative emotions such as fear, depression, boredom, and etc.^[11]^ Being quarantined for a long time can be harmful to mentality and immunity.^[12]^ The involvement of rehabilitation exercises has been reported to relieve symptoms of anxiety and tension, therefore prevent physical weakness, as good mentality can improve the immunity of human body.^[13]^

Moreover, rehabilitation exercises have been added to treatment guidelines for COVID-19 patients in China, as to increase lung function by practicing leisurely activities, such as Tai Chi, Baduanjin, etc.^[14–16]^

However, there is insufficient evidence to show the effectiveness and reliability of using rehabilitation exercises on patients from all severity classifications of COVID-19. We will conduct a meta-analysis and systematic review to evaluate whether effective and practicable rehabilitation can be implemented onto COVID-19 patients from all severity classifications.

## 2 Methods

### 2.1 Review design

This protocol was registered in PROSPERO (CRD42020176972). It refers to the guide book of Preferred Reporting Items for Systematic Reviews and Meta-Analyses Protocols (PRISMA-P).^[17]^ This meta-analysis will be conducted to assess the efficacy and safety of rehabilitation exercises for COVID-19 treatment and relief of the symptoms.

### 2.2 Search strategy

Two independent reviewers will search the following databases, Web of Science, Embase, Cochrane Library, PubMed, Chinese Biomedical Literature Database, Chinese National Knowledge Infrastructure, Chinese Scientific Journal Database, and Wan fang Database, ClinicalTrials, WHO Trials, and Chinese Clinical Trial Registry. Literatures with English or Chinese will be included.

PubMed literature search as following:

#1. Search “COVID-19” [Mesh]
#2. Search (((((((((((((((2019 novel coronavirus infection) OR COVID19) OR coronavirus disease 2019) OR coronavirus disease-19) OR 2019-nCoV disease) OR 2019 novel coronavirus disease) OR 2019-nCoV infection) OR Wuhan coronavirus) OR Wuhan seafood market pneumonia virus) OR COVID19 virus) OR COVID-19 virus) OR coronavirus disease 2019 virus) OR SARS-CoV-2) OR SARS2) OR 2019-nCoV) OR 2019 novel coronavirus
#3. Search #1 OR #2
#4. Search “Exercise Therapy”[Mesh]
#5. Search ((((((((((((Remedial Exercise) AND (Exercise, Remedial)) AND (Exercises, Remedial)) AND (Remedial Exercises)) AND (Therapy, Exercise)) AND (Exercise Therapies)) AND (Therapies, Exercise)) AND (Rehabilitation Exercise)) AND (Exercise, Rehabilitation))) AND (Exercise, Rehabilitation)) AND (Exercises, Rehabilitation)) AND (Rehabilitation Exercises)
#6. Search #4 OR #5
#7. Search # 3 AND #6

### 2.3 Study selection

#### 2.3.1 Type of study

We prioritize randomized controlled trials. Quasi-randomized trials will also be considered. We will assess the risk of bias according to random assignment methods. If it does not mention the method of randomization, we will try to contact author of that literature.

#### 2.3.2 Participants

This study participants will include patients diagnosed with COVID-19. The patients could be any age, sex, or ethnic origin.

Participants will be characterized as 5 categories^[6]^: asymptomatic or presymptomatic infection, mild illness, moderate illness, severe illness, and critical illness according to the inclusion criteria of the retrieved documents.

#### 2.3.3 Type of interventions

We will include the literatures that use rehabilitation exercises as a main variable. Any comparisons between a combined therapy of rehabilitation exercises and other interventions with a therapy of solely using other interventions are also included. No limitation will be used for type of interventions. All the frequencies, durations, and types of rehabilitation exercises will be considered.

#### 2.3.4 Type of comparators

We will include no-treatment, regular treatment, or placebo in the control group.

#### 2.3.5 Primary outcomes

Clinical recovery time and effective rate will be considered the main outcomes. The clinical recovery time is defined as the time until the patient's condition has been better for at least 72 hours after initiating rehabilitation exercises. If the patient's condition gets better, we will consider it into effective rate. Patients can be described as effective when respiratory rate ≤24/min on room air and oxygen saturation >94% on room air. If the literatures describe the effectiveness without same standards, we will contact author of that literature to get further information.

#### 2.3.6 Secondary outcomes

Additional outcomes of patient's condition are as follows:

Changes of patient's condition:

1. Time until COVID-19 RT-PCR tested negative in upper respiratory tract specimen;
2. Time until cough reported as mild or absent;
3. Time until dyspnea reported as mild or absent;
4. Frequency of requirement for supplemental oxygen or noninvasive ventilation;
5. Frequency of requiring respiratory;
6. Incidence of severe cases;
7. Proportion of rehospitalization or admission to ICU;
8. All-cause mortality;
9. Frequency of serious adverse events.

Changes of patient's pulmonary function assessment

1. Forced vital capacity (FVC) and maximal voluntary ventilation;
2. Forced expiratory volume in one second (FEV1) and FEV1/FVC;

Changes of patient's respiratory function assessment

1. Borg Scale^[18]^
2. modified Medical Research Council dyspnoea scale^[19]^

Changes of patient's activity of daily living assessment

1. Barthel Index or modify Barthel Index^[20]^
2. Activities of Daily Living Scale

The secondary results will provide a supplement of rehabilitation exercises affecting on COVID-19. If a new suitable outcome is obtained during literature search, it will be taken into consideration.

#### 2.3.7 Exclusion criteria

Literatures published repeatedly by the same author or with duplicate data; Full data cannot be obtained after contacting with author; Literatures with less than 10 samples in experimental group or control group.

#### 2.3.8 Data extraction

**Figure 1:**
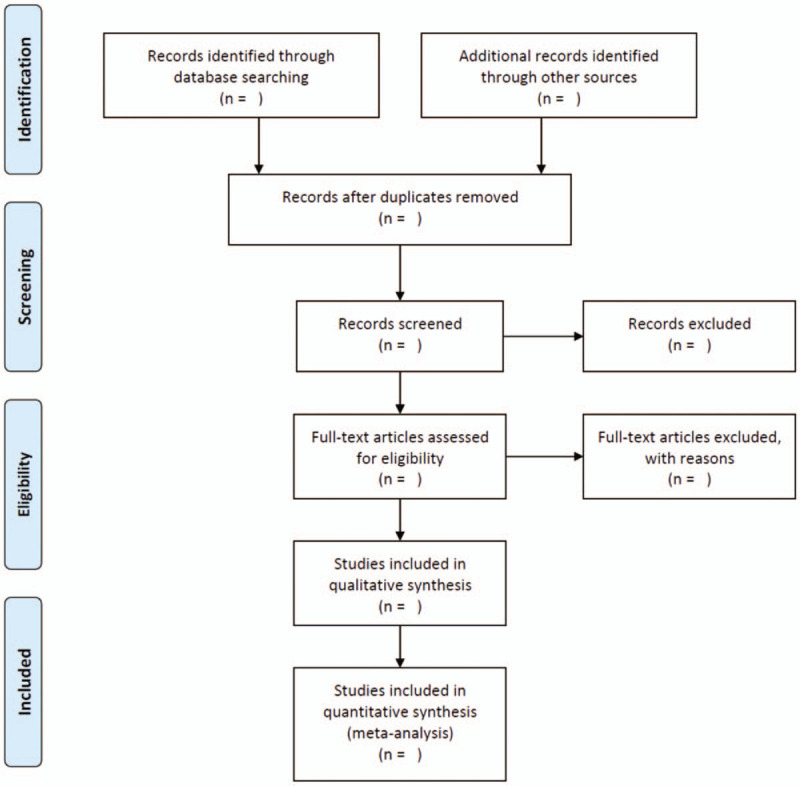
PRISMA flow diagram of the study process. PRISMA =  Preferred Reporting Items for Systematic review and Meta-Analysis.

Endnote X9.3 will be used to manage data screening. The statistical analysis will be completed by RevMan5.2 and Stata/SE 15.1 software. Two review authors (RG, YG) will scan articles through title and abstract independently. Then full text about potentially eligible literatures will be read for selecting appropriate literatures. If 2 independent authors include different literatures, a third expert or the whole group members (CZ, ZS) will take it into discussion. Two main authors independently collect data and present eligible trials characteristics (including the first author, year, patient's condition, experimental group, control group, categories of COVID-19 patients, course of rehabilitation exercises, type of the rehabilitation, and outcomes) in the literatures characteristic form. We will try to contact authors with missing or incomplete data by email. However, if the missing data cannot be obtained, then the study will be excluded from the analysis. We will use PRISMA flow chart to present the process of study selection (Fig. 1).

#### 2.3.9 Risk of bias assessment

According to Cochrane Collaboration's tool for assessing risk of bias,^[21]^ the quality of evidence of the articles included in this review will be assessed by 1 reviewer, and confirmed by a second one. We will discuss when difference exists in risk of bias assessment. Random sequence generation, assignment concealment, blinding of participants and personnel, blinding of outcome assessment, incomplete outcome data, selective reporting and other bias will be assessed to evaluate the bias risks. Afterward, we will provide a chart using green, yellow, red colors and “+”, “−”, “?” to indicate “low risk bias,” “high risk bias,” and “unclear.”

### 2.4 Statistical analysis

#### 2.4.1 Data synthesis

First, we will combine the collected data according to characteristics of eligible trials. For efficiency, we will use fixed effect model to express risk ratio with 95% confidence intervals. On the other hand, the random effect model will be used for continuous outcomes due to clinical heterogeneity. Weighted mean differences or standardized mean differences will be calculated with 95% confidence intervals to assess whether data is statistical significance. Second, we will use *P* value and I^2^ value to test heterogeneity between trial results. If the I^2^>50%, it means that there is heterogeneity among the consolidated data. The statistical package (RevMan 5.2) will be used for data analysis and forest plots in data presentation. When we find obvious heterogeneity among the combined data, we will explore the source of heterogeneity with subgroup analysis, sensitivity analysis and evaluate publication bias.

#### 2.4.2 Subgroup analysis

Subgroup analysis will be carried out to assess the influence of practicing rehabilitation exercises on COVID-19 patients with different levels of severity and different characteristics. It will analyze potential sources of heterogeneity and even out the heterogeneous results for a better evidence, such as evaluating the severity of illness, type of rehabilitation exercises, age, gender, and so on.

### 2.5 Sensitivity analysis

Sensitivity analysis is to assess the bias factors by eliminating each study one at a time. It will calculate sensitivity of each study in the whole project to judge whether an individual study has a significant impact on the results.

### 2.6 Publication bias

Publication bias will be evaluated by using funnel chart and completed by RevMan software. When we have adequate samples, funnel chart will be used to assess bias in small samples.

### 2.7 Quality of evidence

According to grading quality of evidence and strength of recommendations,^[22]^ GRADE system will be used to assess quality of literature with high, moderate, low, or very low level.

## 3 Discussion

Now the ongoing pandemic of COVID-19 has no effective medical cure, therefore it brings an unprecedented challenge to public health in the 21st century. COVID-19 may cause changes in the respiratory system, digestive system,^[23]^ cardiovascular system,^[24]^ etc. The symptoms of respiratory system are the most obvious and serious. Our review is timely since there is an increasing amount of research dedicated to the rehabilitation exercises on respiratory system diseases. Some previous meta-analysis reported that rehabilitation exercises have played a positive role in treating respiratory system diseases,^[25–28]^ and it can improve cardiorespiratory endurance^[29]^^.^ Some trails revealed that rehabilitation exercises can help with pulmonary rehabilitation^[30]^ and COVID-19 is one of the respiratory infectious diseases. In addition, rehabilitation training may improve lung function of COVID-19 patients because of strengthening respiratory muscle, such as intercostal muscles, enthusiastic muscles, abdominal wall muscles, etc. These muscles play an important role in maintaining respiratory function.^[31]^

More and more attentions are being attached to the influence of COVID-19 rehabilitation exercises.^[32,33]^ Suitable rehabilitation may relax muscles, improve respiratory system, and reduce treatment costs, since the outbreak of COVID-19 brought significant medical and economic pressure to many countries. If rehabilitation exercise could help with COVID-19 treatment or enhance respiratory function and prevent its damage, it will be benefiting patients and relieving much medical pressure. However, there is no evidence to verify its effectiveness and safety for COVID-19 patients at present.

It is difficult to prove that COVID-19 patients from all severity levels to receive rehabilitation exercises, our systematic review and meta-analysis will be conducted to collect existing trails data based on these different levels of classification. It will provide an overview of the application of rehabilitation exercises for adjuvant treatment for COVID-19 patients. Strengths and limitations will be highlighted during identifying evidence. The data extraction and risk of bias assessment will be completed by 2 researchers independently to improve the quality of evidence. Furthermore, the protocol was registered PROSPERO. It will follow the guideline of Cochrane^[21]^ and PRISMA statement.^[17]^ Limitations will mainly be originated from different clinical condition and different basic treatment on COVID-19 patients. However, funnel plots, subgroup analyses, and sensitivity analysis will be conducted to explore the sources of heterogeneities. The results of this systematic review and meta-analysis may establish a better approach to adjuvant treatment for COVID-19 and analyze application of rehabilitation exercises on COVID-19 patients with different levels of severity.

## Author contributions

**Conceptualization:** Renjun Gu, Ziyun Li, Zhiguang Sun, Yihuang Gu.

**Methodology:** Renjun Gu, Senlei Xu, Yihuang Gu.

**Project administration:** Renjun Gu, Ziyun Li, Zhiguang Sun.

**Supervision:** Zhiguang Sun, Yihuang Gu.

**Writing – original draft:** Renjun Gu, Senlei Xu, Ziyun Li.

**Writing – review & editing:** Renjun Gu, Zhiguang Sun, Yihuang Gu.

## References

[1] Rothan HA Byrareddy SN The epidemiology and pathogenesis of coronavirus disease (COVID-19) outbreak. J Autoimmun 2020;109:102433. 32113704 10.1016/j.jaut.2020.102433 PMC7127067

[2] Lu R Zhao X Li J Genomic characterisation and epidemiology of 2019 novel coronavirus: implications for virus origins and receptor binding. Lancet 2020;395:565–74. 32007145 10.1016/S0140-6736(20)30251-8 PMC7159086

[3] Contini C Di Nuzzo M Barp N The novel zoonotic COVID-19 pandemic: an expected global health concern. J Infect Dev Ctries 2020;14:254–64. 32235085 10.3855/jidc.12671

[4] Paudel S Dangal G Chalise A The coronavirus pandemic: what does the evidence show? J Nepal Health Res Counc 2020;18:1–9. 32335585 10.33314/jnhrc.v18i1.2596

[5] Guo YR Cao QD Hong ZS The origin, transmission and clinical therapies on coronavirus disease 2019 (COVID-19) outbreak—an update on the status. Mil Med Res 2020;7:11. 32169119 10.1186/s40779-020-00240-0 PMC7068984

[6] COVID-19 Treatment Guidelines Panel. Coronavirus Diseases 2019 (COVID-19) Treatment Guidelines. National Institutes of Health. Accessed date 20 May, 2020. Available at https://www.covid19treatmentguidelines.nih.gov/.

[7] Kaminsky LA Arena R Ellingsen Ø Cardiorespiratory fitness and cardiovascular disease —the past, present, and future. Prog Cardiovasc Dis 2019;62:86–93. 30639135 10.1016/j.pcad.2019.01.002

[8] Lavie CJ Ozemek C Carbone S Sedentary behavior, exercise, and cardiovascular health. Circ Res 2019;124:799–815. 30817262 10.1161/CIRCRESAHA.118.312669

[9] Fletcher GF Landolfo C Niebauer J Promoting physical activity and exercise: JACC health promotion series. J Am Coll Cardiol 2018;72:1622–39. 30261965 10.1016/j.jacc.2018.08.2141

[10] Lake MA What we know so far: COVID-19 current clinical knowledge and research. Clin Med (Lond) 2020;20:124–7. 32139372 10.7861/clinmed.2019-coron PMC7081812

[11] Brooks SK Webster RK Smith LE The psychological impact of quarantine and how to reduce it: rapid review of the evidence. Lancet 2020;395:912–20. 32112714 10.1016/S0140-6736(20)30460-8 PMC7158942

[12] Nussbaumer-Streit B Mayr V Dobrescu AI Quarantine alone or in combination with other public health measures to control COVID-19: a rapid review. Cochrane Database Syst Rev 2020;4:CD013574. 32267544 10.1002/14651858.CD013574 PMC7141753

[13] Jiménez-Pavón D, Carbonell-Baeza A, Lavie CJ. Physical exercise as therapy to fight against the mental and physical consequences of COVID-19 quarantine: Special focus in older people [published online ahead of print, 2020 Mar 24]. Prog Cardiovasc Dis. 2020. 10.1016/j.pcad.2020.03.009 PMC7118448 32220590

[14] Yang F Liu N Wu JY Zhonghua Jie He He Hu Xi Za Zhi 2020;43:E004. 32023687 10.3760/cma.j.issn.1001-0939.2020.0004

[15] Xia W Hua Q Wang G standard for rehabilitation diagnosis and treatment of COVID-19 with integration of traditional Chinese and Western medicine. Rehabil J 2020;30:85–92.

[16] Feng C Cui H Yu H Novel coronavirus pneumonia recovery period comprehensive intervention plan of TCM expert's guidance (Draft). Beijing Chin Med 2020;39:102–4.

[17] Shamseer L Moher D Clarke M Preferred reporting items for systematic review and meta-analysis protocols (PRISMA-P) 2015: elaboration and explanation [published correction appears in BMJ. 2016 Jul 21;354:i4086]. BMJ. 2015;350:g7647. Published 2015 Jan 2. doi: 10.1136/bmj.g7647. 10.1136/bmj.g7647 25555855

[18] Bray NW Smart RR Jakobi JM Exercise prescription to reverse frailty. Appl Physiol Nutr Metab 2016;41:1112–6. 27649859 10.1139/apnm-2016-0226

[19] Rajala K Lehto JT Sutinen E mMRC dyspnoea scale indicates impaired quality of life and increased pain in patients with idiopathic pulmonary fibrosis. ERJ Open Res 2017;3: 00084-2017. 10.1183/23120541.00084-2017 PMC5731772 29255720

[20] Huybrechts KF Caro JJ The Barthel Index and modified Rankin Scale as prognostic tools for long-term outcomes after stroke: a qualitative review of the literature. Curr Med Res Opin 2007;23:1627–36. 17559756 10.1185/030079907x210444

[21] Higgins JPT Savović J Page MJ, JAC S: Chapter 8: Assessing risk of bias in a randomized trial. Cochrane Handbook for Systematic Reviews of Interventions version 6.0 (updated 2019): Cochrane; 2019.

[22] Atkins D Best D Briss PA Grading quality of evidence and strength of recommendations. BMJ 2004;328:1490. 15205295 10.1136/bmj.328.7454.1490 PMC428525

[23] Wong SH Lui RN Sung JJ Covid-19 and the digestive system. J Gastroenterol Hepatol 2020;35:744–8. 32215956 10.1111/jgh.15047

[24] Zheng YY Ma YT Zhang JY COVID-19 and the cardiovascular system. Nat Rev Cardiol 2020;17:259–60. 32139904 10.1038/s41569-020-0360-5 PMC7095524

[25] Radtke T Nevitt SJ Hebestreit H Physical exercise training for cystic fibrosis. Cochrane Database Syst Rev 2017;11:CD002768. 29090734 10.1002/14651858.CD002768.pub4 PMC6485991

[26] Jenkins AR Gowler H Curtis F Efficacy of supervised maintenance exercise following pulmonary rehabilitation on health care use: a systematic review and meta-analysis. Int J Chron Obstruct Pulmon Dis 2018;13:257–73. 29391784 10.2147/COPD.S150650 PMC5768431

[27] Lahham A McDonald CF Holland AE Exercise training alone or with the addition of activity counseling improves physical activity levels in COPD: a systematic review and meta-analysis of randomized controlled trials. Int J Chron Obstruct Pulmon Dis 2016;11:3121–36. 27994451 10.2147/COPD.S121263 PMC5153296

[28] Wu W Liu X Wang L Effects of Tai Chi on exercise capacity and health-related quality of life in patients with chronic obstructive pulmonary disease: a systematic review and meta-analysis. Int J Chron Obstruct Pulmon Dis 2014;9:1253–63. 25404855 10.2147/COPD.S70862 PMC4230171

[29] Rezende Barbosa MPDC Oliveira VC Silva AKFD Effectiveness of functional training on cardiorespiratory parameters: a systematic review and meta-analysis of randomized controlled trials. Clin Physiol Funct Imaging 2018;38:539–46. 28752947 10.1111/cpf.12445

[30] Polkey MI Qiu ZH Zhou L Tai Chi and pulmonary rehabilitation compared for treatment-naive patients with COPD: a randomized controlled trial. Chest 2018;153:1116–24. 29625777 10.1016/j.chest.2018.01.053

[31] Liu K Zhang W Yang Y Respiratory rehabilitation in elderly patients with COVID-19: a randomized controlled study. Complement Ther Clin Pract 2020;39:101166. 32379637 10.1016/j.ctcp.2020.101166 PMC7118596

[32] Kiekens C Boldrini P Andreoli A Rehabilitation and respiratory management in the acute and early post-acute phase. “Instant paper from the field” on rehabilitation answers to the Covid-19 emergency. Eur J Phys Rehabil Med 2020;Online ahead of print. 10.23736/S1973-9087.20.06305-4 32293817

[33] Ceravolo MG, De Sire A, Andrenelli E, et al. Systematic rapid “living” review on rehabilitation needs due to covid-19: update to March 31st 2020. Eur J Phys Rehabil Med. 2020; Online ahead of print. 10.23736/S1973-9087.20.06329-7 32316718

